# Rapid Deployment Aortic Valve Replacement: Valve of Choice in Patients With a Left Circumflex Anomaly?

**DOI:** 10.1016/j.atssr.2024.11.015

**Published:** 2024-12-17

**Authors:** Julia von der Linden, Polyxeni Vlachea, Florian Herrmann, Sergey Belyaev, Gerd Juchem, Sven Peterss, Christian Hagl, Alexey Dashkevich

**Affiliations:** 1 Department of Cardiac Surgery, Ludwig Maximilian University of Munich, Munich, Germany; 2 Munich Heart Alliance, Partner Site German Centre for Cardiovascular Disease (DZHK), Munich, Germany; 3 Department of Cardiac Surgery, University of Leipzig, Leipzig, Germany

## Abstract

We present a successful application of rapid deployment surgical aortic valve replacement in a patient with an anomalous origin of the left circumflex coronary artery, which originated from the right coronary ostium. The patient presented with cardiac decompensation, with resting dyspnea and angina pectoris. Imaging revealed high-grade aortic valve insufficiency, a reduced left ventricular ejection fraction, an aneurysm of the ascending aorta (58 mm), and relevant stenoses of the coronary arteries. She underwent rapid deployment surgical aortic valve replacement, after careful dissection of the left circumflex artery and replacement of the ascending aorta, proximal arch, and bypasses. Her left ventricular ejection fraction improved postoperatively, and there were no complications during the inpatient course and at 1-year follow-up.

The origin of the left circumflex coronary artery (LCX) from the right coronary ostium is the most common coronary anomaly and is often an incidental finding. The course of the LCX artery around the noncoronary sinus of the aortic valve can cause challenges in both surgical aortic valve replacement (SAVR) and transcatheter aortic valve replacement.^1^ Rapid deployment (RD) valves are associated with simplification of implant procedures, reduced intraoperative times, and excellent hemodynamics.^2^ We report a case of successful implantation of an RD valve in a patient with severe aortic regurgitation, an aortic aneurysm, multiple coronary stenoses, and an atypical origin of the LCX from the RCA ostium.

Our patient, a 73-year-old woman (46 kg, 163 cm) presented with progressive dyspnea at rest (New York Heart Association functional class IV) for several weeks, as well as typical angina (Canadian Cardiovascular Society class IV). At presentation, she showed signs of cardiac decompensation, including pleural effusions. A thoracic computed tomographic scan, performed to rule out pulmonary artery embolism, revealed an ascending aortic aneurysm with a diameter of up to 58 mm, without significant dilation of the aortic root (39 mm × 40 mm). The patient was admitted to our hospital (Department of Cardiac Surgery, LMU of Munich) for completion of diagnostic testing and evaluation of surgical options. Transthoracic echocardiography revealed severe aortic valve regurgitation, no aortic valve stenosis, and impaired left ventricular function (left ventricular ejection fraction, 34%). The left ventricle was dilated, with an end-diastolic diameter of 49 mm and an end-systolic diameter of 35 mm. Coronary angiography revealed 2-vessel coronary disease with significant stenoses of the left anterior descending (LAD) coronary artery and the right coronary artery (RCA). We were surprised to see that the left circumflex (LCX) coronary artery had an anomalous origin from the right coronary ostium (Figure 1).

**Figure 1:**
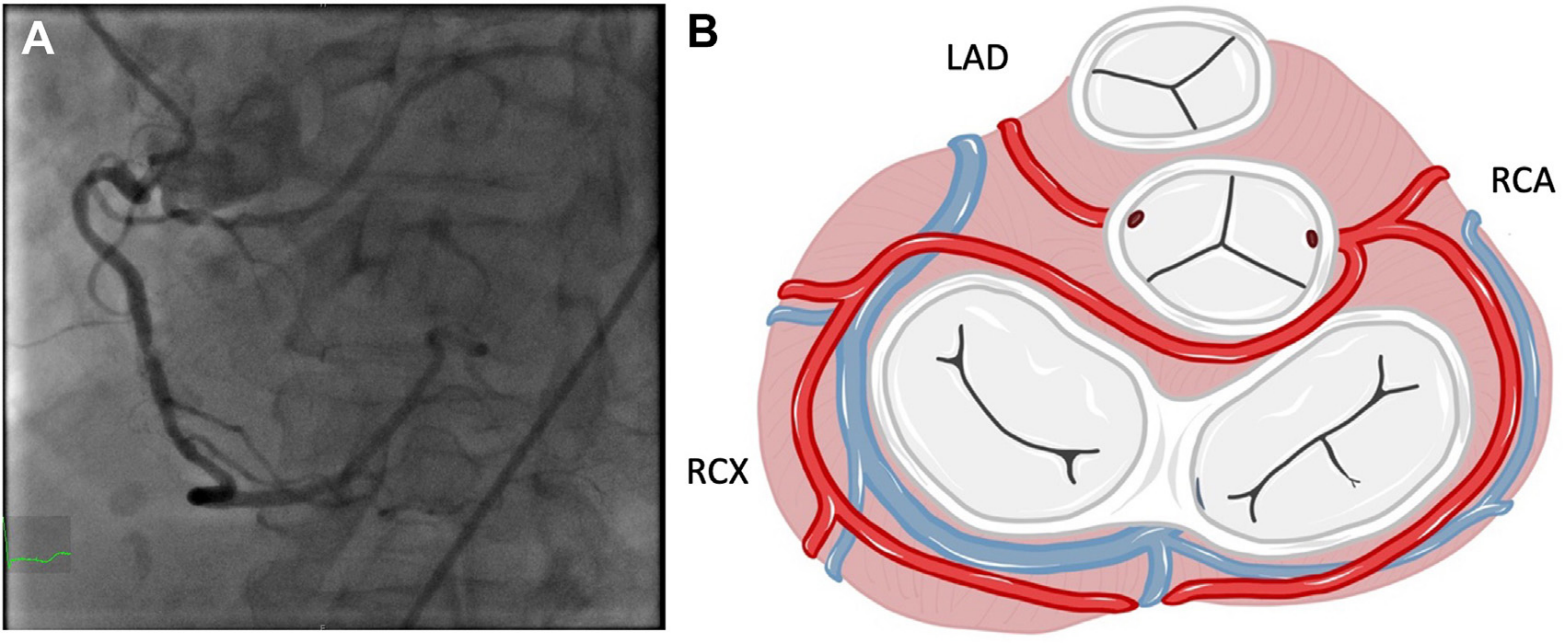
of the left circumflex coronary artery from the right coronary ostium with a retroaortic course as seen in (A) coronary angiography and (B) a schematic representation. (LAD, left anterior descending coronary artery; RCA, right coronary artery; RCX, right circumflex.)

The patient underwent a combined cardiac procedure with RD SAVR, supracoronary replacement of the ascending aorta, hemiarch replacement in hypothermic circulatory arrest (25.2 °C with antegrade cerebral perfusion), and coronary artery bypass grafting of the LAD artery and RCA through a median sternotomy. Cardiopulmonary bypass was established by cannulation of the right atrium and the ascending aorta. Myocardial protection was accomplished by antegrade cold (4 °C) Bretschneider cardioplegia through an aortic root cardioplegia cannula. After a transverse aortotomy, inspection revealed a tricuspid aortic valve with no calcifications. The atypical course of the LCX coronary artery under the level of the aortic ring coursing clockwise around the noncoronary sinus was confirmed by careful preparation of the vessel. The LCX artery was completely detached from the bordering left atrial and aortic walls. After removal of the aortic valve cusps and sizing of the aortic ring, a 21-mm Edwards INTUITY Elite Valve (Edwards Lifesciences) was implanted after careful annular suture placement at the cusp nadirs (indexed effective orifice area >0.85 cm^2^/m^2^). We took great care to ensure that the mobilized LCX artery would not be impaired by the deployed prosthesis (Figure 2). As a result of the combined procedure, the decision was made to use an RD prosthesis to reduce the prolonged intraoperative times. This decision was based on the very good experience with this prosthesis and the excellent postoperative results obtained in previous patients.

**Figure 2:**
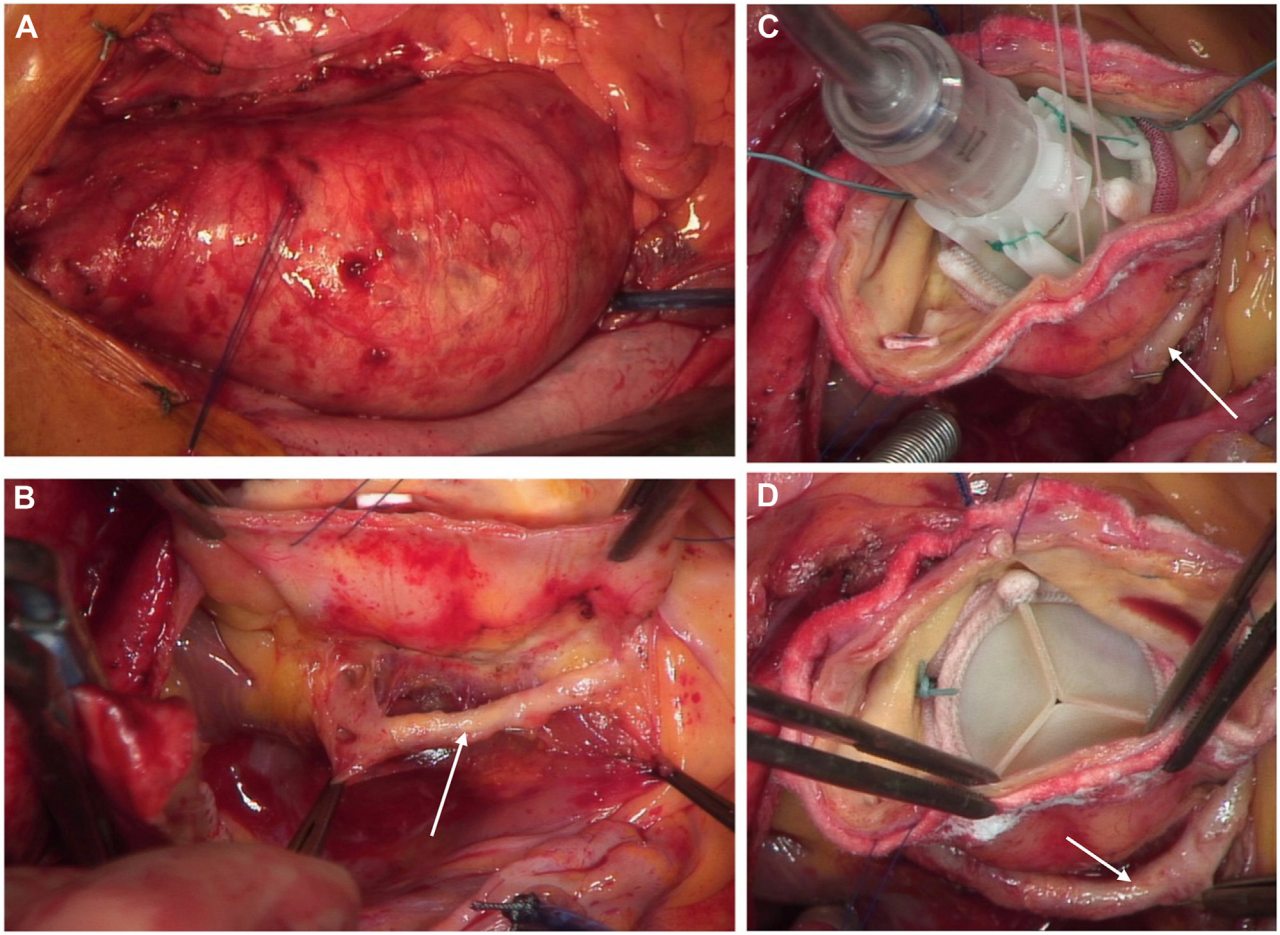
Intraoperative images during the procedure. (A-C) course of the aberrant left circumflex coronary artery (LCX) around the noncoronary sinus. (D) Rapid deployment valve after implantation, with no signs of strain to the LCX. The arrows in B–D indicate the LCX.

Furthermore, we performed supracoronary replacement of the ascending aorta, as well as hemiarch replacement with an Ante-Flo vascular graft (Vascutek, Terumo Aortic) in hypothermic circulatory arrest and bypass grafting of the LAD artery and the RCA. The LAD artery was revascularized through the left internal mammary artery, and the RCA was revascularized through a saphenous vein graft. The vein graft was proximally anastomosed with the aortic prosthesis. The aortic cross-clamp time was 131 minutes, the cardiopulmonary bypass time was 198 minutes, and the circulatory arrest time was 19 minutes. Postoperatively, laboratory values, as well as echocardiographic and electrocardiographic findings, showed no signs of myocardial ischemia. The postoperative course was uneventful, and the patient was discharged 28 days after surgery (the extended postoperative hospital stay was related to geriatric issues causing delayed mobilization). Transthoracic echocardiography performed shortly before discharge revealed a bioprosthesis with no signs of paravalvular leakage; the mean transvalvular gradient was 9 mm Hg and the peak gradient was 17 mm Hg. The ejection fraction was improved, with only minor impairment. The patient has reported good postoperative results, with increased performance.

## Comment

We describe the use of RD SAVR to improve workflow during aortic valve replacement in a patient with an aberrant LCX artery. The occurrence of coronary artery anomalies is rare; angiographic series have suggested a prevalence of 0.6% to 3.1%. Aberrant origin of the LCX artery from the right coronary ostium or the right sinus of Valsalva has been described most frequently (34%-58%). These are congenital anomalies with different variations, which are usually benign and discovered incidentally. However, one-fifth of all such anomalies can lead to life-threatening conditions such as arrhythmias, syncope, sudden death, or myocardial infarction.^1^^,^^3^, ^4^, ^5^ Aberrant artery compression from implanted material in the aortic root has the potential to provoke such sequelae.

Subsequently, careful surgical planning is key in such cases.^2^ Our preferred surgical strategy is complete mobilization of the aberrant coronary artery and choice of an ideal valve prosthesis with reduced pledget and suture requirement. We thus opt to perform RD SAVR in such cases. When a conventional valve is implanted, strain on the aberrant artery may be caused by compression through the suture ring, traction from circumferential annular sutures, and compression from implanted pledgets. Use of RD SAVR has the benefit that no pledgets and only 3 guiding sutures are required, thereby reducing the risk of strain to the LCX artery. The subannular location of the valve’s fixation mechanism, the stent frame, further reduces the risk of strain because it subsequently has no contact with the aberrant artery.

In our experience, LCX artery aberrance is rare. When a cardiac surgeon is confronted with this situation in aortic valve surgery, we believe that the RD aortic valve is a feasible option with a reduced risk of complications when performed by a surgeon experienced in RD SAVR. Furthermore, the introduction of an RD valve into a major combined procedure such as the one presented here can relevantly reduce aortic cross-clamp times.
